# Anti-Inflammatory, Antidiabetic, and Antioxidant Properties of Extracts Prepared from Pinot Noir Grape Marc, Free and Incorporated in Porous Silica-Based Supports

**DOI:** 10.3390/molecules29133122

**Published:** 2024-06-30

**Authors:** Mihaela Deaconu, Anil Abduraman, Ana-Maria Brezoiu, Nada K. Sedky, Simona Ioniță, Cristian Matei, Laila Ziko, Daniela Berger

**Affiliations:** 1 Faculty of Chemical Engineering and Biotechnologies, National University of Science and Technology Politehnica Bucharest, 1-7 Gheorghe Polizu Street, 011061 Bucharest, Romania; mihaela.deaconu@upb.ro (M.D.); ana_maria.brezoiu@upb.ro (A.-M.B.); simona.ionita05@gmail.com (S.I.); 2 CAMPUS Research Institute, National University of Science and Technology Politehnica Bucharest, 060042 Bucharest, Romania; 3 Department of Biochemistry, School of Life and Medical Sciences, University of Hertfordshire, Hosted by Global Academic Foundation, R5 New Garden City, New Administrative Capital, Cairo 11835, Egypt; nadasedky22@gmail.com (N.K.S.); l.adel@gaf.edu.eg (L.Z.)

**Keywords:** porous silica, grape marc, phenolic extract, anti-inflammatory properties, antioxidant activity, antidiabetic activity, extract encapsulation

## Abstract

This study presents properties of hydroethanolic extracts prepared from Pinot Noir (PN) grape pomace through conventional, ultrasound-assisted or solvothermal extraction. The components of the extracts were identified by HPLC. The total content of polyphenols, flavonoids, anthocyanins, and condensed tannins, as well as antioxidant activity and α-glucosidase inhibitory activity of extracts were evaluated using UV-vis spectroscopy. All extracts were rich in phenolic compounds, proving a good radical scavenging activity. The extract obtained by conventional extraction at 80 °C showed the best α-glucosidase inhibitory activity close to that of (-)-epigallocatechin gallate. To improve the chemical stability of polyphenols, the chosen extract was incorporated in porous silica-based supports: amine functionalized silica (MCM-NH_2_), fucoidan-coated amine functionalized silica (MCM-NH_2_-Fuc), MCM-41, and diatomite. The PN extract exhibited moderate activity against Gram-positive *S. aureus* (MIC = 156.25 μg/mL) better than against Gram-negative *E. coli* (MIC = 312.5 μg/mL). The biocompatibility of PN extract, free and incorporated in MCM-NH_2_ and MCM-NH_2_-Fuc, was assessed on RAW 264.7 mouse macrophage cells, and the samples showcased a good cytocompatibility at 10 µg/mL concentration. At this concentration, PN and PN@MCM-NH_2_-Fuc reduced the inflammation by inhibiting NO production. The anti-inflammatory potential against COX and LOX enzymes of selected samples was evaluated and compared with that of Indomethacin and Zileuton, respectively. The best anti-inflammatory activity was observed when PN extract was loaded on MCM-NH_2_-Fuc support.

## 1. Introduction

Since wine is the most widespread alcoholic beverage in the world, wine production generates a large amount of grape pomace byproduct, millions of tons each year according to the International Organization of Vine and Wine. This is rich in phenolic compounds that can be recovered by extraction, a way to reduce the environmental impact of the wine making process [1,2]. The bioactive compounds derived from the grape pomace are valuable substances that have attracted attention due to their biological effects, like anti-inflammatory, neuroprotective, antioxidant, cardioprotective, antibacterial, antiproliferative [3].

Research on the application of plant extracts, as antioxidants, antibacterial, and anti-inflammatory agents in cosmetics, nutraceuticals, or foods is constantly growing [4,5,6]. The radical scavenging activity of extracts depends on their chemical profile, hence the development of functional products based on extract formulations requires efficient methods of extracting natural compounds from different sources, especially from abundant vegetal residues, such as grape marc.

Conventional solvent extraction is widely applied, mainly due to its effectiveness, ease of application, and lack of expensive equipment, but it requires long extraction time, high temperature, and a large volume of solvent, which may lead to the alteration of the extract properties because some natural compounds are not stable under these conditions. However, this method is widely accepted as an efficient technique for recovering bioactive substances [7]. Microwave-assisted extraction and ultrasound-assisted extraction significantly reduce the process time and often the temperature, thus reducing energy consumption and making the process greener [8]. High hydrostatic pressure extraction has been proven to be efficient for flavonoid extraction from propolis [9] or caffeine from green tea leaves [10], resulting in a higher extraction yield than conventional extraction due to the increase in solubility of organic compounds with increasing pressure. Pressurized liquid extraction is usually carried out at high pressure (40–200 bar) and temperature (50–250 °C) in static or dynamic conditions [11].

One of the drawbacks of using extract components as natural antioxidants or antimicrobial agents is related to their low chemical and thermal stability [12,13]. To overcome these issues, nanoencapsulation of phenolic compounds could be employed to enhance their stability and this has been demonstrated in several reports [14,15,16,17].

This study aimed to develop effective processes for the extraction of phenolic compounds from grape pomace of Pinot Noir cultivar from Murfatlar (Black Sea region, Romania). We report the influence of the extraction method (ultrasound-assisted, extraction under inert gas pressure, and conventional method) and temperature on the chemical profile of hydroethanolic extracts. The extracts were fully characterized concerning their chemical profile. To delay the degradation process of extract components and to improve the biological properties, extracts with good antioxidant activity were encapsulated in porous supports, such as mesoporous silica pristine, and functionalized with amino groups (MCM-NH_2_), fucoidan-coated amino functionalized silica (MCM-NH_2_-Fuc), and diatomite.

Mesoporous silica is known for its high chemical stability, low toxicity, biocompatibility, and textural parameters, such as high pore volume, and therefore, it is widely used as support for biologically active substances. The pore array structure of these nanoparticles allows the tuning of the interactions between the silica support and encapsulated organic molecules, offering the possibility to control the adsorption and desorption processes [18].

Lately, biopolymers have received special attention due to the current trend of natural resource valorization, but also for their features, such as biodegradability, sustainability, and abundance in nature. Natural polysaccharides are known for their antioxidant, antitumor, anti-inflammatory, antiviral, and antimicrobial activity. Hence, they can be used for biomedical purposes [19]. Fucoidan is a natural polysaccharide extracted from brown seaweed (e.g., *Fucus vesiculosus*), which consists of fucose units linked alternately by α-(1,3) and α-(1,4) glycosidic bonds with sulfate groups [20]. Diatomite consists of porous siliceous skeletons of diatom frustules and it can be considered a biomaterial as it exhibits non-toxicity, high adsorption capacity, and thermal and chemical stability. It has been used as a carrier in drug delivery systems [21], adsorbent material [22], or support in energy storage applications [23].

The cytotoxicity and NO production inhibitory effect of a selected extract, both free and incorporated in amine functionalized mesoporous silica and fucoidan-coated silica, was tested on the mouse macrophage cell line (RAW264.7) at two sample doses of treatment, 10 μg/mL and 100 μg/mL. Also, the anti-inflammatory potential of prepared extract, free and encapsulated, was evaluated in vitro using COX and lipoxygenase (LOX) enzyme inhibition assays.

## 2. Results

### 2.1. Characterization of Extracts

#### 2.1.1. Spectrometric Determinations for Extracts

The waste material, dried grape marc from the production of Pinot Noir wine in 2020, was used for obtaining extracts by (i) conventional method at two temperatures, 40 °C—PN(C) and 80 °C—PN(R), (ii) ultrasound-assisted extraction (USAE) at 40 °C—PN(US), and (iii) under argon pressure (5 atm) in a solvothermal reactor, at 80 °C—PN. In the case of the conventional method, extraction in three steps of 60 min with solvent replacement were applied, while for USAE, shorter stages of 15 min were used. For all extracts, the solvent was 1:1 (*v*/*v*) ethanol–water mixture and the ratio of grape pomace/solvent volume was 1/18 g/mL. Once the extraction was finished, the solid was separated by filtration and dried. The extraction yield varied in the 15.3–17.7% range, the highest value being achieved by extraction under argon pressure, while the lowest value was obtained for USAE. A better recovery of phenolic compounds was obtained when the extraction was carried out at higher temperature, 80 °C (16.8%) compared to 40 °C (16.1%).

**Figure 1:**
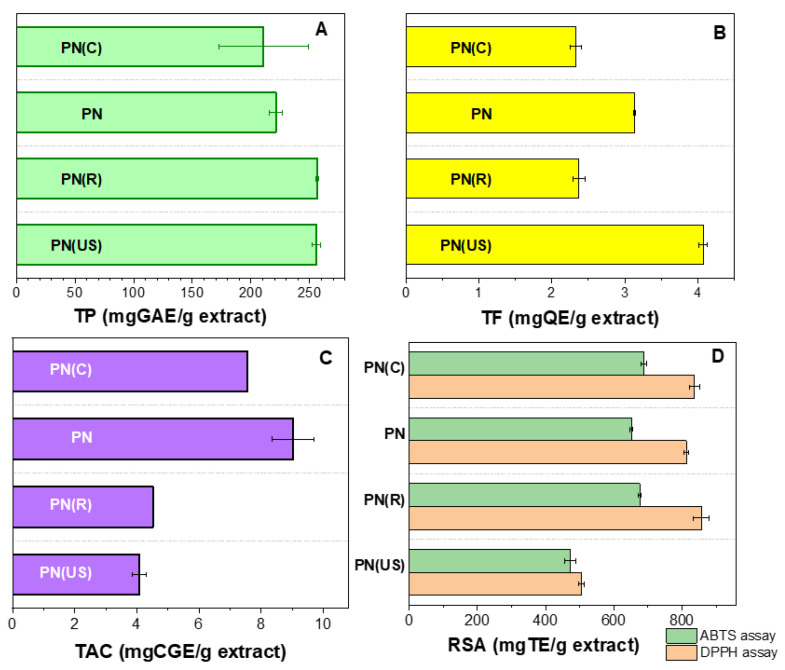
Spectrometric determinations for the prepared extracts: TP as gallic acid equivalents, GAE (**A**); TF as quercetin equivalents, QE (**B**); TAC as cyanidin-glucoside equivalents, CGE (**C**); RSA as Trolox equivalents, TE (**D**).

Characterization of extracts was carried out by spectrometric determinations (three replicates) of total polyphenolic (TP, Figure 1A), total flavonoid (TF, Figure 1B), total anthocyanins (TAC, Figure 1C) contents, as well as radical scavenging activity—DPPH and ABTS assays (Figure 1D) based on the methods described in our previous papers [16,17].

All extracts had high content of phenolic compounds that varied in the range of 33.87–43.16 mgGAE/g dried marc. Among the extracts obtained at 40 °C, the sample prepared through USAE led to a higher TP index than the conventional method (39.25 versus 33.87 mgGAE/g dried marc), while in the case of samples obtained at 80 °C, better results were obtained by conventional extraction than through solvothermal method (43.16 versus 39.11 mgGAE/g dried marc). All TP values are higher than our previous reported results for extracts prepared from grape pomace of other cultivars. With respect to the extraction of flavonoids, USAE proved to be the most efficient method (4.07 mgQ/g extract), while the least effective technique was the conventional extraction regardless of the temperature (2.33 at 40 °C and 2.37 mgQ/g extract at 80 °C). Among phenolic compounds, anthocyanins are unstable compounds, sensitive to pH and UV radiation [24]. One can notice that the highest TAC value was obtained when the extraction was performed in argon protective atmosphere—PN extract (9.30 mgCGE/g extract), followed by conventional method at 40 °C (7.55 mgCGE/g extract). Ultrasound and higher temperature (80 °C) has a negative effect on the anthocyanin’s extraction (4.08 and 4.52 mgCGE/g extract for PN(US) and PN(R), respectively). However, PN extracts have lower amounts of anthocyanins than that of extracts prepared from *Feteasca Neagra* (FN) and *Cabernet Saugvinon* (CS) cultivars from the same winemaking producer (12.38 and 13.06 mgCGE/g extract, respectively) [16].

All extracts had good antioxidant activity in the range of 505.55–856.86 mg TE/g extract (DPPH assay) and 473.78–689.02 mg TE/g extract (ABTS method), the lowest values being obtained for the extract prepared by USAE. The conventional and pressurized extracts exerted a much better radical scavenging activity assessed through both assays (812.47–856.86 mg TE/g extract -DPPH and 651.80–689.09 m mg TE/g extract -ABTS).

The condensed tannins content (CTC) expressed as catechin equivalents (CE) was evaluated through the vanillin method [25]. PN and PN(R) extracts had 265.09 ± 4.41 mg CE/g plant (46.92 ± 0.78 mg CE/g marc) and 206.30 ± 1.17 mg CE/g extract (34.66 ± 0.20 mg CE/g marc), respectively.

#### 2.1.2. Chemical Composition of PN Grape Pomace Extracts

Eight polyphenols were determined in Pinot Noir extracts through reverse phase high performance liquid chromatography (RP-HPLC) with diode array detector (Table 1). The chromatograms can be found in Supplementary Material, Figure S2. Higher content of phenolics from catechins class was observed for extracts prepared at 80 °C (11.505–18.722 mg catechin hydrate/g extract and 8.772–13.218 mg (-) epicatechin/g extract) than that those obtained at 40 °C (11.505–12.784 mg catechin hydrate/g extract and 8.772–9.700 mg (-) epicatechin/g extract). From standard compounds available for analysis, cyanidin, malvidin, pelargonidin, and delphinidin, only delphinidin was identified and quantified in the range of 0.401–0.453 mg/g extract in Pinot Noir extracts, except the sample prepared by USAE (0.080 mg/g extract). From the class of phenolic acids, gallic, protocatechuic, vanillic, and syringic acids were identified in similar quantities in all Pinot Noir extracts, while from flavanols group, rutin hydrate was determined in the range of 0.499–0.559 mg rutin/g extract, the highest amount being found in PN(R) extract (Table 1). No stilbenes were found in our Pinot Noir extracts.

#### 2.1.3. Antidiabetic Potential via α-Glucosidase Inhibitory Activity Assessment

The antidiabetic potential of Pinot Noir extracts was evaluated by α-glucosidase inhibitory activity assay [26] using (-)-epigallocatechin gallate (EGCG) as positive control. Catechins, especially EGCG are known for their antidiabetic potential [27]. IC_50_ value for positive control was 9.23 ± 0.61 µg/mL. Half-maximal inhibitory concentrations (IC_50_) for PN and PN(R), which were rich in catechin derivatives, are 13.52 ± 2.85 and 9.70 ± 1.16 µg/mL, respectively. One can notice a very close IC_50_ value of PN(R) extract (9.70 ± 1.16 µg/mL) to the value determined for EGCG, while PN was slightly less effective as an inhibitor to α-glucosidase activity.

#### 2.1.4. Antimicrobial Activity of PN Extract

The antimicrobial activity of PN extract was tested against two standard bacterial strains, Gram-positive *Staphylococcus aureus* (ATCC 25923) and Gram-negative *Escherichia coli* (ATCC 25922). The extract was more potent against Gram-positive strain, with a minimum inhibitory concentration (MIC) of 156.25 μg/mL, while against *E. coli* the extract exhibited a weaker antibacterial activity (MIC = 312.5 μg/mL).

### 2.2. Incorporation of PN Extract in Porous Supports

For the incorporation of PN extract, the following porous silica-based supports were used: aminopropyl functionalized mesoporous silica denoted MCM-NH_2_, fucoidan-coated aminopropyl functionalized silica labelled MCM-NH_2_-Fuc, pristine MCM-41 silica, and diatomite.

#### 2.2.1. Supports Characterization

Pristine and aminopropyl functionalized silica (obtained from pristine silica through post-synthesis approach) were analyzed by small-angle X-ray diffraction (XRD, Miniflex 2, Rigaku Holdings Corporation, Tokyo, Japan) to evaluate the type of porous framework, FTIR spectroscopy (Bruker Tensor 27, Bruker Corporation Optik GmbH, Bremen, Germany) to evidence the presence of amine groups chemically bound on the silica surface, thermogravimetric analysis (TGA, Netzsch STA 2500 Regulus, Selb, Germany) for determination of organic groups content, and scanning electron microscopy (SEM, Tescan Vega 3 LMH microscope, Brno, Czech Republic) to investigate the samples’ morphology.

**Figure 2:**
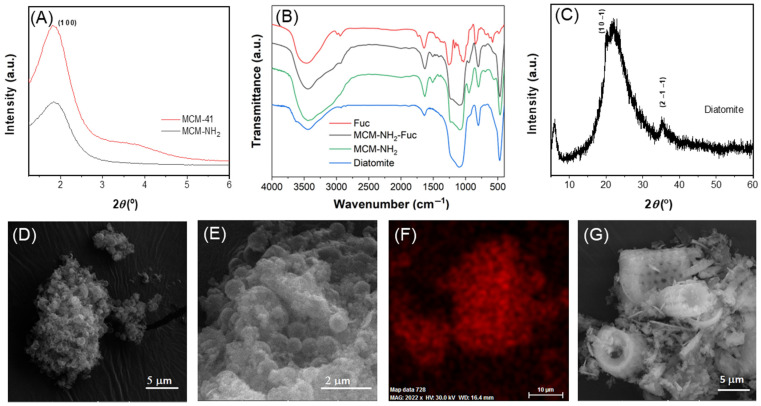
Small-angle X-ray diffraction patterns of MCM-41-type support (**A**); FTIR spectra of diatomite, MCM-NH_2_, MCM-NH_2_-Fuc, and fucoidan denoted Fuc (**B**); Wide-angle XRD analysis of diatomite (**C**); SEM micrographs of MCM-NH_2_ sample (**D**), MCM-NH_2_-Fuc support (**E**), sulfur (red) mapping of MCM-NH_2_-Fuc surface (**F**), and diatomite (**G**).

Figure 2A shows the presence of the (100) diffraction peak, characteristic of MCM-41-type silica, for MCM-41 and MCM-NH_2_ samples, with a lower intensity in the case of the functionalized support. Because of the presence of organic groups, the degree of ordering of the mesopore network diminished after functionalization. One can note in the FTIR spectra (Figure 2B) of all supports, the vibrations of silica (800 cm^−1^ and 1150 cm^−1^—the symmetric and asymmetric stretching vibrations of Si-O-Si bonds, 460 cm^−1^ and 900 cm^−1^—the deformation and stretching vibrations, respectively of silanol groups), as well as the bands characteristic of the functional groups in the case of the MCM-NH_2_ and MCM-NH_2_-Fuc materials (the deformation band of -NH_2_ groups at 1506 cm^−1^ and in 2980–2875 cm^−1^ domain, the stretching vibrations of C-H bonds of aliphatic methylene groups). In the FTIR spectra of all porous supports, one can also observe a large band of medium intensity attributed to the stretching vibrations of -OH groups (3200–3400 cm^−1^), as well as the deformation band of physio-adsorbed water at 1630 cm^−1^ [28]. The stretching vibrations of S=O groups from fucoidan molecules are at 1120 cm^−1^ and 1270 cm^−1^, but they overlap silica bands. However, in the spectrum of MCM-NH_2_-Fuc, the characteristic band of C-O-S bonds can be seen at 845 cm^−1^ (Figure 2B) [29].

SEM characterization evidenced that MCM-NH_2_ material has spherical particles (Figure 2D), while MCM-NH_2_-Fuc support (Figure 2E) present more agglomerated primary nanoparticles than MCM-NH_2_. The elemental mapping of the MCM-NH_2_-Fuc surface shows a uniform dispersion of polymer on silica particles, as sulfur is well distributed on the silica surface (Figure 2F).

Diatomite powder consists of individual frustules containing rows of pores (interstriae). Their silica cell walls consist of two overlapping thecae [30]. The structure of diatomite support was structurally investigated by X-ray diffraction and its morphology by SEM. XRD analysis demonstrated that silica cell walls are mainly amorphous (Figure 2C). However, a tridymite phase (in small quantity) with hexagonal symmetry (P6_3_/22 space group) was evidenced in XRD patterns for diatomite, through the presence of (1 0 −1) and (2 −1 −1) Bragg reflections characteristic of tridymite (ICDD-83-1949). It is worth mentioning the diffraction peak, which corresponds to *d* = 1.497 nm, can be attributed to the ordered pore array of diatomite frustules. SEM investigation of diatomite evidenced mainly the shape of frustules and their rows of pores. The diatomite frustules have an inner diameter of 5–7 μm and a length in the range of 9–11 μm (Figure 2G).

**Figure 3:**
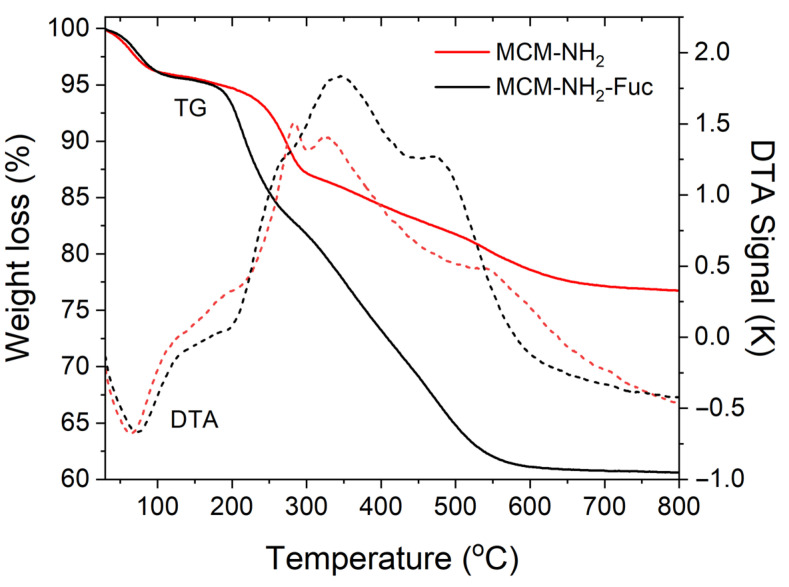
TGA-DTA curves for MCM-NH_2_ and MCM-NH_2_-Fuc supports.

Thermogravimetric analysis (TGA) was employed to find out the content of 3-aminopropyl moieties chemically bonded on the silica surface and the amount of polymer that coated the functionalized silica particles. Based on TGA, the molar ratio of SiO_2_/amine groups is 1/0.23 in the case of MCM-NH_2_ material, and the amount of fucoidan of MCM-NH_2_-Fuc support is 7.49% wt. (Figure 3), taking into account that the polymer alone has a residue of 20.83% wt. at 600 °C.

#### 2.2.2. Characterization of PN-Loaded Silica-Based Supports

To obtain PN-loaded materials, the wet impregnation technique was employed using a 50% ethanol aqueous solution of extract (15 mg/mL) and the corresponding support dried under low pressure (3 mbar) for at least 4 h. After homogenization of the support with extract solution, the solvent was removed at low pressure (3 mbar) in a desiccator connected to a vacuum pump (Vacuubrand MD 1C, Wertheim, Germany). It was considered a theoretical extract content of 25% wt. (for functionalized materials) or 30% wt. (for unfunctionalized supports) in the resulting extract-loaded material.

- Thermal analysis

**Figure 4:**
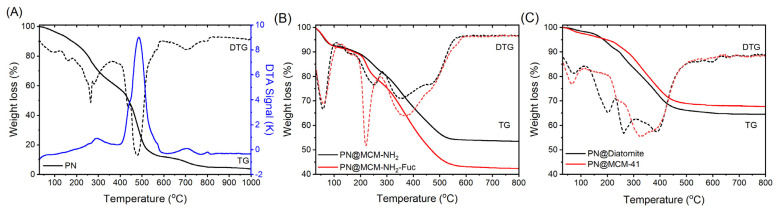
TGA-DTG curves of PN (**A**), PN@MCM-NH_2_ and PN@MCM-NH_2_-Fuc (**B**); as well as for PN@MCM-41 and PN@Diatomite (**C**).

The amount of bioactive compounds entrapped in the support pores was evaluated by thermogravimetric analysis considering the solid residue of PN extract (5.13% wt.) in Figure 4A. The solid residue of PN extract (after calcination at 800 °C) was analyzed by energy dispersive X-ray spectroscopy and SEM that evidenced the presence of only potassium and carbon (Supplementary Materials, Figure S2). In the case of PN extract, the mass loss in the temperature range of 30–110 °C is attributed to the solvent removal, followed by decomposition of extract components with lower stability in the 110–357 °C temperature domain. Combustion of organic compounds occurs in the temperature range of 357–592 °C, while the formation and crystallization of inorganic compounds takes place in the 592–750 °C temperature domain, being accompanied by a small weight loss assigned to the oxidation of carbonaceous residue of organic compounds (Figure 4A). The content of PN extract of PN@MCM-NH_2_-Fuc, computed from TGA curves was 25% wt., while in the case of PN@MCM-NH_2_, was 28% wt. (Figure 4A). In the DTG curves (Figure 4B), one can notice similar thermal behavior of PN@MCM-NH_2_ and PN@MCM-NH_2_-Fuc, but a higher weight loss, as a result of the thermal decomposition not only of the functional groups bound on the silica surface, but also of the polymer. The combustion of extract components, as well as organic moieties from supports occurs from 110 °C, up to 530 °C as the two effects can be observed on the DTG curves (Figure 4B). In the case of materials containing diatomite, PN@Diatomite, and MCM-41, PN@MCM-41, the extract content was 29.7% wt. and 29.8% wt., respectively (Figure 4C). The thermal decomposition of phenolic compounds from extract in the case of PN@MCM-41 samples start at 238 °C and is completed at 500 °C, while for PN@Diatomite, it begins at 257 °C and ends at the same temperature as in the case of PN@MCM-41 (Figure 4C). The weight loss up to 238 °C can be assigned to diatomite.

- Wide-angle X-ray powder diffraction

**Figure 5:**
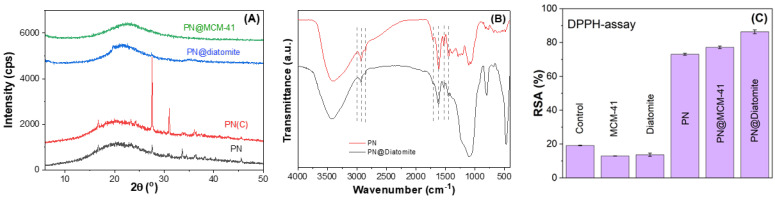
XRD patterns of PN, PN(C), PN@diatomite, and PN@MCM-41 (**A**); FTIR spectra of PN@diatomite in comparison with that for PN extract (**B**); radical scavenging activity of PN@MCM-41 and PN@Diatomite in comparison with that of PN free extract and corresponding supports using DPPH solution as control after 12 months of storage at 4 °C (**C**).

The extract-loaded materials were investigated through wide-angle XRD, which demonstrated that phenolic substances from extract were confined into the pores of support since they are in an amorphous state in PN-loaded supports (Figure 5A). When comparing the XRD pattern of the extract obtained at high pressure, PN, with that of the sample prepared at low temperature by conventional method, PN(C), the same crystalline compounds are present in both extracts, but their crystallinity is higher in the PN(C) sample (Figure 5A).

- FTIR spectroscopy

In the FTIR spectra of PN extract, one can observe the specific bands of catechins at 1619 cm^−1^, 1278 cm^−1^, and 1107 cm^−1^, which are the most abundant phenolic compounds in the extract and overlapped with intense vibrations of silica matrix. In the FTIR spectra of PN-loaded supports, one can see the stretching bands of hydroxyl groups in the 3200–3600 cm^−1^ domain, and at 1442 cm^−1^, the vibration attributed to the deformation band of OH groups superimposed the stretching vibrations of C-O bonds characteristic of phenols. One can notice the stretching bands of C-H bonds of aliphatic methylene groups at 2930 cm^−1^, 2853 cm^−1^, and that of C-H bonds belonging to a phenyl ring at 3010 cm^−1^, as well as the band from 1713 cm^−1^ assigned to C=O bonds (Figure 5B) [31,32].

- Radical scavenging activity

The radical scavenging activity (RSA) of PN extract. free and incorporated in diatomite or pristine MCM-41 silica, was evaluated in vitro by the DPPH method applied to solid samples developed by our group [16], after 12 months of storage at 4 °C. We demonstrated that MCM-41 silica is a good matrix for phytocompound encapsulation, offering a preservation of their radical scavenging properties [16]. This was the reason to compare RSA of the extract incorporated in diatomite with that of the PN extract entrapped in MCM-41 matrix using a DPPH assay. Both PN@Diatomite and PN@MCM-41 samples showed a better RSA than PN alone after one year of storage (Figure 4C). It was found that PN@Diatomite had the highest RSA value, which means that diatomite protected the bioactive compounds from chemical degradation.

### 2.3. Biological Evaluation of PN Extract and PN-Loaded Supports

#### 2.3.1. Cytotoxicity and NO Production Inhibitory Effects

**Figure 6:**
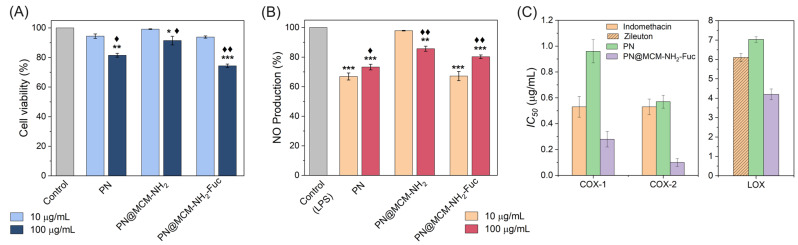
Cell viability and NO production inhibitory effects in RAW 264.7 cells. (**A**) Cellular viability after 72 h of exposure to the test samples compared to the Control group (Untreated cells). (**B**) NO generation in cells treated with the different test samples for 1 h before the addition of LPS and induction of inflammation as compared to the Control (cells that received LPS only). The symbol (*) indicates significant difference from the control, while the symbol (♦) refers to significant difference from 10 µg/mL concentration of the test sample. The use of each symbol for once, twice, and thrice indicate *p*-values < 0.05, <0.01, and <0.001, respectively. (**C**) Anti-inflammatory potential against COX-1 and COX-2 in comparison with Indomethacin, and LOX compared to Zileuton. Data displayed as the average of triplicates ± standard deviation (SD).

RAW 264.7 cells were incubated for 72 h with two different concentrations (10 and 100 μg/mL) of each of the test agents, then cellular viability was determined using an SRB assay. The entire set of the test samples did not exhibit any significant cytotoxic activity against RAW 264.7 cells at the low concentration (10 μg/mL), rendering this concentration very safe to the cells. Meanwhile, the application of the higher concentration (100 μg/mL) of any of the test samples displayed a significant cytotoxic activity against RAW 264.7 cells as compared to control group (untreated) or even the group treated with the lower concentration, 10 μg/mL (Figure 6A).

The two different concentrations of the samples (10 and 100 μg/mL) were added to RAW 264.7 cells an hour prior to the induction of inflammation by LPS (1 μg/mL). The supernatants were collected and assayed for the generated NO using Griess reagent. Pre-treatment with both 10 and 100 μg/mL concentrations of the majority of samples resulted in a significant reduction in NO production in LPS-stimulated RAW 264.7 cells (Figure 6B). The only exception was that PN@MCM-NH_2_ caused a significant reduction in the production of NO at the high concentration (100 μg/mL), while the low concentration (10 μg/mL) did not cause any effect on NO production. It is noteworthy to mention that the low concentration (10 μg/mL) was significantly more efficient than the high concentration (100 μg/mL) for both PN and PN@MCM-NH_2_-Fuc at reducing the NO production (Figure 5B). The exposure to PN at a concentration of 10 and 100 μg/mL caused about 33% and 27% inhibition of NO production, respectively. The application of PN@MCM-NH_2_-Fuc at a concentration of 10 μg/mL caused about 33% inhibition of NO production, while at a concentration of 100 μg/mL it caused about 20% inhibition of NO production. Therefore, the low concentration (10 μg/mL) of both PN and PN@MCM-NH_2_-Fuc could be very promising to reduce the inflammation by inhibiting NO production, while preserving the normal cell’s viability.

#### 2.3.2. Anti-Inflammatory Activity against Cyclooxygenase and Lipoxygenase Enzymes

The two test samples (PN and PN@MCM-NH_2_-Fuc) that caused the highest NO inhibitory effects were further examined for their ability to inhibit COX-1 and COX-2 enzymes. An anti-inflammatory drug, indomethacin, was used as reference for the obtained results. The half maximal inhibitory concentration (IC_50_) was calculated for each of the test samples against both COX-1 and COX-2. The current findings indicated that both PN and PN@MCM-NH_2_-Fuc formulations resulted in a significant inhibition of COX-2 at an estimated IC_50_ value of 0.57 and 0.1 μg/mL, respectively. The IC_50_ value of PN against COX-2 enzyme was almost equal to that of the reference drug (Indomethacin). Moreover, the IC_50_ of the PN@MCM-NH_2_-Fuc against COX-2 enzyme was about 5-fold less than that of PN or Indomethacin, thereby indicating a much higher potency of PN@MCM-NH_2_-Fuc against COX-2 enzyme that surpassed all other test samples (Figure 6C). Both PN and PN@MCM-NH_2_-Fuc formulations were more selective for COX-2 enzyme as indicated by selectivity index (SI = IC_50_ COX-1/IC_50_ COX-2) of 1.7 and 2.8, respectively. As such, they have a promising potential of being used therapeutically as COX-2-specific anti-inflammatory drugs that would suppress inflammation while having minimal side effects and preserving the stomach mucosa [33]. The PN@MCM-NH_2_-Fuc formulation has a particular importance as it possesses higher inhibitory effects, especially for COX-2 enzyme.

The two samples (PN and PN@MCM-NH_2_-Fuc), as well as the reference drug, Zileuton, were assessed for their ability to inhibit LOX enzyme and IC_50_ values were calculated for all the examined samples (Figure 6C). The IC_50_ of PN was 7.03 μg/mL which was somewhat comparable to that of Zileuton (IC_50_ of 6.10 μg/mL) with no remarkable difference. The formulation PN@MCM-NH_2_-Fuc has shown to be the most potent inhibitor of LOX as indicated by the lowest IC_50_ (4.20 μg/mL) compared to the other two (reference drug and PN).

## 3. Discussion

Usually, *Vitis vinifera* L. pomace is used as a supplement in animal feed or for production of organic fertilizers. As a waste that is produced in large quantities in a relatively short period of time (August–October), rich in antioxidants, its valorization through antioxidant extraction reduces its impact on the environment [34]. In recent years, extracts containing phenolic compounds have received attention, as the need for bioactive compounds that are employed as antioxidants or antibacterial agents is constantly increasing. Extracts containing antioxidants can find many uses in extending the shelf life of foods through preventing lipid oxidation or protein damage. However, they have certain disadvantages concerning the chemical instability over time. To overcome this drawback, different formulations containing phenolic substances were proposed [6,16,17]. Extracts can also be utilized for health benefits like anti-inflammatory, antidiabetic, or antimicrobial properties by being incorporated in nutraceuticals or supplements [35,36,37].

Herein we report properties of extracts obtained from Pinot Noir grape marc in different conditions. We developed a new method for polyphenol extraction using a solvothermal reactor. Usually, high temperature and pressure are used in pressurized liquid extraction to exceed the boiling point of the solvent, which often has a negative impact on the stability of the bioactive compounds. To avoid this, an additional pressure of 5 atm of argon was introduced in the reactor and then the solvent was heated at 80 °C. The high pressure improves the solubility of organic compounds and reduces that of inorganic ones. Thus, using this extraction method, an enhanced extraction yield was obtained (17.68% wt.).

All Pinot Noir extracts reported in this study exhibited a very good radical scavenging activity with results in the range of 505.55–856.86 mg TE/g extract in the case of DPPH method and 473.78–689.02 mg TE/g extract for ABTS assay (Figure 1), values which are higher than that reported by us for Cabernet Sauvignon and Feteasca Neagra ethanolic extracts (344 and 119 mg TE/g extract, respectively—DPPH method) [16]. The good antioxidant activity could be correlated with their high polyphenol amount. Regarding the extraction technique, USAE led to a lower content of anthocyanins in extract, while the conventional method yielded a worse recovery of flavonoids. Our extracts exerted up to 2.7 times higher radical scavenging activity (153.42–460.36 μmol TE/g grape marc—ABTS assay) than that reported by Pertuzatti et al. for an extract prepared from Bordo grape marc in methanol–water–formic acid mixture (171.03 μmol TE/g grape marc) [1]. The high RSA values are consistent with the big TP index of Pinot Noir extracts, especially for the samples obtained at 80 °C, (43.16 and 39.11 mgGAE/g dried marc for PN and PN(R), respectively), which are higher than that obtained by us for red grape pomace ethanolic extracts from CS and FN (24.33 and 27.10 mgGAE/g dried marc) [16] or by Tsali et al. [38] for extract prepared from red pomace in 24% aqueous ethanol solution (26.44 mgGAE/g dried marc).

PN and PN(R) extracts were chosen for the determination of the condensed tannins index due to their high content of phenolics from catechin class. PN sample had the highest condensed tannins index (46.92 ± 0.78 mg catechin/g marc). However, our extract had two-fold lower content of condensed tannins than the hydroethanolic extracts prepared at room temperature by Balea et al. (93.41 ± 1.34 mg epicatechin/g dried marc) from the same cultivar, but harvested from Mures County, Romania, in 2015 [39].

Concerning the chemical composition of hydroethanolic Pinot Noir extracts prepared at 80 °C, they have a high content of catechin hydrate and (-) epicatechin, 11.505–18.722 mg and 8.772–13.218 mg per gram extract, amounts that are higher than that noticed for ethanolic extracts from CS and FN cultivars from the same region (0.184 ± 0.005 mg catechin hydrate/g extract and 2.666 ± 0.018 (-) epicatechin/g extract for CS extract and 2.270 ± 0.005 mg (-) epicatechin/g extract for FN extract, respectively) [16]. From the anthocyanins class, only delphinidin was quantified in all PN extracts ranging from 0.080 to 0.453 mg/g extract, unlike the case of our CS ethanolic extract for which pelargonidin was identified (0.606 mg/g extract) [16]. The acidic ethanolic extract from PN cultivar from Araucanía region (Chile) was rich in malvidin-3-glucoside, while delphinin-3-glucoside was found in the lowest concentration [24]. In the case of Pinot Noir wine (from Canterbury, New Zealand) the most abundant anthocyanin pigment was malvidin3-glucoside, followed by peonidin-3-O-glucoside [40].

Phenols, such as catechins, anthocyanins, and tannins, have been recognized for their antidiabetic activity acting through reducing blood glucose levels, specifically by inhibiting digestive enzymes such as α-amylase and α-glucosidase. Galloylated catechins better inhibit the activity of α-amylase than non-galloylated catechins. This was the reason for choosing EGCG as a positive control in our experiments for determination of the antidiabetic potential via α-glucosidase inhibition activity [41,42,43]. Our PN(R) and PN samples exerted great α-glucosidase inhibition activity (IC_50_ in the range of 9.70–13.52 μg/mL extract), values which are close to the positive control, EGCG, and much higher than that exhibited by the best extract reported from Tannat grape pomace from Uruguay, (the hydro-alcoholic extract obtained in formic acid medium—IC_50_ = 888.5 ± 79.3 μg/mL) [44] or by red Cabernet Sauvignon grape seeds extract complex with a derivative of β-cyclodextrin (1035 μg/mL) [45]. When comparing the values for IC_50_ of PN and PN(R) extracts with that determined for other extracts well known for their antidiabetic activity, lower values were obtained. For instance, for *Vaccinium myrtillus* extracts, the following IC_50_ values were determined: 35.81 ± 2.51 µg/mL for ethanolic extract prepared by USAE [46], 30.46 ± 0.98 µg/mL for aqueous extract [47], or 2.53 ± 0.21 mg/mL and 0.29 ± 0.02 mg/mL for aqueous and hydroethanolic extract, respectively [48].

The PN extract had higher antibacterial activity against *S. aureus* (MIC = 0.156 mg/mL) than against *E. coli* (MIC = 0.312 mg/mL), which is consistent with the data published by Krasteva et al. [49] for 70% aqueous ethanolic extract prepared from Pinot Noir grape seed extract. However, they obtained a lower MIC against *S. aureus* (0.12 mg/mL) and higher value against *E. coli* (0.50 mg/mL). In another study, for 80% aqueous acetonic extracts from four Virginia (U.S.A.) grape marc varieties, MIC values against *Listeria monocytogenes* ATCC 7644 and *S. aureus* ATCC 29213 were in the range of 4.69–18.8 mg/mL and 40.6–250 mg/mL, respectively, and no antibacterial activity was recorded against *E. Coli* ATCC 3510 [50]. Sateriale et al. reported bacteriostatic and bactericidal effects for their hydroethanolic extract from Aglianico (*Vitis vinifera* L.) grape pomace against *S. aureus* ATCC 25923 and *E. coli* ATCC 25922 with higher MIC values (15 mg/mL and 40 mg/mL, respectively) than that of our extracts [51].

Incorporating extracts into inert supports usually improves their chemical stability over time, and we have demonstrated in previous works [16,52,53] that mesoporous silica nanoparticles can be used for this purpose. Therefore, we chose to incorporate the PN extract prepared under inert gas pressure because the resulting extract was the richest in anthocyanins (1.597 ± 0.119 mgCGE/g pomace) and tannins (46.92 ± 0.78 mg CE/g pomace). In this study, amine functionalized mesoporous silica, MCM-NH_2_, and fucoidan-coated amine-functionalized silica, MCM-NH_2_-Fuc, were proposed as matrices for PN incorporation for the development of formulations with anti-inflammatory properties, which could be further included as ingredients in nutraceuticals. Also, in this work, diatomite was considered as support for PN extract and compared with pristine mesoporous silica. Recently, diatomite was employed for a slow-release fertilizer and for water retention [54]. In the last 15 years an increase of productivity was observed for various crops when diatomite was used as fertilizer [55]. Artem et al. proposed ecological fertilizers comprising grape marc besides algae, invertebrate shells, etc. [56].

FTIR spectroscopy highlighted the presence of catechins, besides other phenolic compounds, while the amorphous nature of encapsulated extract into porous silica-type supports suggested that all biologically active substances from extract are nanoconfined in their porous structure. Based on in vitro spectrophotometric determination of radical scavenging activity of extract-loaded materials, it was found that PN@Diatomite had the highest RSA value, even higher than PN@MCM-41, which means that not only mesoporous silica, but also diatomite is able to protect the bioactive compounds from chemical degradation.

In the current work, we chose 10 and 100 µg/mL concentrations for evaluation of biocompatibility, as well as NO production inhibitory effects of PN extract alone and incorporated in MCM-NH_2_ or MCM-NH_2_-Fuc supports on normal RAW 264.7 cells to compare our previous results on formulations based on mesoporous silica containing hydroethanolic grape marc extract from Mamaia cultivar [17] or bilberry fruit extracts [52]. All tested samples have demonstrated no observable cytotoxic activity against normal RAW 264.7 cells, especially when applied at the low concentration (10 μg/mL), thereby keeping the normal cells intact and secure. Likewise, a study that included *Vitis vinifera* L. *Pinot Noir* grape cane reported no noticeable cytotoxic activity for the whole extract against the MRC-5 normal lung fibroblast cells [57]. As such, the safety of the extracts can be guaranteed. Nitric oxide promotes blood pressure reduction and interacts with superoxide anions with peroxynitrite formation causing DNA damage [58]. The anti-inflammatory activity of both PN and PN@MCM-NH_2_-Fuc at the low concentration (10 μg/mL) has been evidenced by their distinguished ability to inhibit NO production as compared to the control group. It was reported that a treatment on LPS induced inflammation in gingival fibroblast cell lines with white grape pomace extract of 100 µg/mL significantly reduced IL-8 levels, while for red pomace extract, a concentration of 200 µg/mL was required for similar anti-inflammatory effects [59]. Fariña et al. showed that Tannat grape pomace extracts inhibited TNF-α mediated NF-κB activation and IL-8 production in human colon cancer HT-29 cells [60].

In a similar context, a former study revealed that pre-treatment with grape pomace extracts from Romania could decrease the inflammation-induced oxidative stress in rats [61]. The difference is that in this study, the low concentration (10 μg/mL) of both PN and PN@MCM-NH_2_-Fuc exhibited higher anti-inflammatory activity against LPS-induced inflammation in RAW 264.7 cells as compared to the high concentration (100 μg/mL), while Balea et al. showed that the extract reduced turpentine oil-induced inflammation in a dose-dependent manner in rats [61].

The anti-inflammatory activity of the free extract, PN, as well as PN@MCM-NH_2_-Fuc were further warranted by their ability to inhibit COX and LOX enzymes. Such enzymes are widely known as proinflammatory enzymes owing to their implication in the inflammatory process [62]. The potency of both PN and PN@MCM-NH_2_-Fuc at the inhibition of COX enzymes were comparable to the reference drug, Indomethacin. The PN@MCM-NH_2_-Fuc formulation was ranked the topmost potent inhibitor with the highest selectivity towards COX-2. This entails that PN@MCM-NH_2_-Fuc would suppress inflammation while having minimal side effects and preserving the stomach mucosa [33]. Chiavaroli et al. observed that aqueous grape pomace extract from Montepulciano d’Abruzzo (Italy) and catechin, one of the main components of the extract, were effective in upregulation of COX-2 gene expression in HypoE22 cells treated also with hydrogen peroxide at dose of treatment [63].

LOX enzyme is a mediator of the second metabolic pathway that produces eicosanoids. Leukotriene B4 is the end-product of the 5-LOX pathway, and it is involved in the pathophysiology of various inflammatory conditions, including atherosclerosis [64]. Thereby, the inhibition of LOX is considered an important aspect of reducing the inflammatory process. Our findings revealed that the ability of the newly formulated PN@MCM-NH_2_-Fuc to inhibit LOX exceeded that of the FDA approved LOX inhibitor, Zileuton. This in turn provides cumulative evidence for the prominent anti-inflammatory activity of PN@MCM-NH_2_-Fuc sample.

To sum up, the evaluation of anti-inflammatory potential of PN extract incorporated in fucoidan coated amine functionalized silica support, MCM-NH_2_-Fuc, showed higher efficiency than the free extract. Thus, both PN extract and PN@MCM-NH_2_-Fuc formulation at low concentration (10 μg/mL) could reduce the inflammation through inhibiting NO production without affecting normal cells’ viability. Moreover, the PN@MCM-NH_2_-Fuc sample exerted better anti-inflammatory activity against COX and LOX enzymes than the free extract or the reference drugs, Indomethacin and Zileuton, respectively. Thus, the IC_50_ for PN@MCM-NH_2_-Fuc against COX-2 enzyme (0.10 ± 0.03 μg/mL) was about 5 times lower than that of PN (0.57 ± 0.05 μg/mL) or Indomethacin (0.53 ± 0.06 μg/mL). Both PN and PN@MCM-NH_2_-Fuc samples were selective for COX-2 enzyme, the selectivity index being 1.7 and 2.8, respectively. The IC_50_ value for PN@MCM-NH_2_-Fuc is lower than for bilberry fruit extract (0.97 μg/mL) or for bilberry fruit extract incorporated in silica functionalized with thiol groups (0.69 μg/mL) [52].

Regarding the anti-inflammatory potential against the LOX enzyme, the incorporation of the PN extract into the MCM-NH_2_-Fuc support had a better effectiveness (IC_50_ = 4.20 μg/mL) than free PN extract (IC_50_ = 7.03 μg/mL) or Zileuton (IC_50_ = 6.10 μg/mL).

## 4. Materials and Methods

### 4.1. Preparation of Pinot Noir Extracts

All chemicals used for extracts characterization and support synthesis are listed in Supplementary Material. In this study, to obtain hydroethanolic extracts, air-dried grape marc from Pinot Noir wine production in 2020 (Murfatlar, Constanța County, Romania) was used. The solvent for extraction was 1/1 water/absolute ethanol (*v*/*v*) and the vegetal material/solvent ratio was 1/18 g/mL. The conventional extraction was carried out at 40 °C (PN(C) extract) and 80 °C (PN(R) extract), respectively, in three stages of 60 min, while the ultrasound-assisted extraction (Bandelin Sonorex Digitec, Berlin, Germany) was done at 40 °C, in three steps of 15 min resulting in the extract denoted PN(US). The extract labelled PN was obtained under 5 atm of argon pressure, at 80 °C for 3 h using a solvothermal reactor with a temperature controller (Roth Karlsruhe WRX 2000, Frederikssund, Denmark). After removal of exhausted vegetal material, the extracts were dried at 40 °C using a rotary evaporator (DLab RE100-Pro, DLAB Scientific Co., LTD., Beijing, China).

### 4.2. Characterization of Pinot Noir Extracts

To characterize the extracts, spectrometric determinations (Shimadzu UV-1800 spectrophotometer, Kyoto, Japan) of total polyphenols, flavonoids, anthocyanins content, as well as of radical scavenging activity (DPPH and ABTS methods) were carried out as described elsewhere [16,17]. The composition of the extracts was analyzed through reverse phase high-performance liquid chromatography (Shimadzu Nexera X2, Kyoto, Japan) with photodiode array detector (SPD-M30A, Shimadzu, Kyoto, Japan). The method for polyphenol identification and quantification using 24 standard compounds (see Supplementary Material) was described in references [16,17].

*Determination of condensed tannins content.* For condensed tannins determination, vanillin method [25] was used. Briefly, 50 µL sample of 2.5 mg/mL concentration, 1.5 mL vanillin methanolic solution (4%), and 0.75 mL concentrated hydrochloric acid were mixed and then the resulting mixture was kept 20 min., at room temperature. The solution absorbance was measured at 500 nm. Experiments were conducted in triplicate.

*Assessment of antidiabetic potential* via *α-glucosidase inhibitory activity.* For antidiabetic potential evaluation via α-glucosidase inhibitory activity assay, 150 µL sample with various concentrations were added to 450 µL phosphate buffer solution 10 mM pH 6.8 and 150 µL α-glucosidase 0.5 units/mL solution. The mixture was incubated at 37 °C for 15 min, followed by the addition of α-glucosidase substrate solution, 150 µL *p*-nitrophenol-α-d-glucopyranoside 5 mM, and incubation at 37 °C for another 15 min. The reaction was completed with 600 µL Na_2_CO_3_ 200 mM aqueous solution and the absorbance was determined at 405 nm. Half maximal inhibitory concentrations (IC_50_) were determined by linear regression based on the following equations: y = 4.028·x − 4.465; R^2^ = 0.9778 (10–25 µg/mL concentration domain) for PN sample and y = 6.389·x − 11.975; R^2^ = 0.9792 (2–15 µg/mL concentration domain) for PN(R) extract. Experiments were carried out in three replicates.

*Determination of antibacterial potential.* 20 mg/mL PN extract was prepared and tested against *S. aureus* (ATCC 25923) and *E. coli* (ATCC 25922) strains. Serial dilutions were carried out from the stock solution. Overnight cultures were prepared from each strain and used in the diffusion agar technique to determine the MIC values. Each well diameter was 6 mm and 100 μL of each concentration was added to the well for testing the antibacterial effect.

### 4.3. Obtaining and Characterization of Porous Supports

Pristine MCM-41 and aminopropyl functionalized silica nanoparticles, MCM-NH_2_, were synthesized according to the procedures reported previously [51]. Specific surface area of 888 m^2^/g and 410 m^2^/g, total pores volume (measured at 0.99 relative pressure) of 0.99 cm^3^/g and 0.58 cm^3^/g, and average pore diameter (according to the Density Functional Theory) of 4.2 nm and 3.8 nm for MCM-41 and MCM-NH_2_, respectively, were computed based on nitrogen adsorption-desorption isotherms (Micromeritics TriStar II Plus, Norcross, GA, USA) (Supplementary Material, Figure S3). MCM-NH_2_-Fuc material was prepared by coating 88.8 mg MCM-NH_2_ sample with 8.8 mL of aqueous fucoidan (from *Fucus vesiculosus*) solution (5 mg/mL). The resulting mixture was maintained under magnetic stirring (180 rpm) for 90 min and then the solvent was evaporated at 40 °C by using a rotary evaporator. Diatomite (diatomaceous earth) was purchased from Sigma-Aldrich (Merck Group, Darmstadt, Germany) and used without purification.

### 4.4. Biological Evaluation of PN Extract Free and Incorporated, in Porous Supports

*Anti-inflammatory assay in mouse macrophage cells.* Mouse macrophage cell line (RAW264.7) was purchased from Nawah Scientific Inc. (Cairo, Egypt). The cells were maintained under standard culture conditions, i.e., DMEM media augmented with streptomycin (100 mg/mL), penicillin (100 units/mL), and 10% of heat-inactivated fetal bovine serum at 37 °C in a 5% CO_2_ incubator with humidified atmosphere. The cells were left in the incubator for an hour, then 10 and 100 μg/mL of each of the test agents were deposited in the corresponding wells. Cell viability of RAW264.7 cells was determined following three days of exposure to the test agents using an SRB assay as described in our previous work [52].

The amounts of NO produced were estimated in cell culture supernatants following a formerly described protocol [65]. Cellular suspension containing 10^5^ RAW264.7 cells was placed in every well of a 96-well plate and the plate was kept in the incubator for one day. Thereafter, wells were treated with two different concentrations of the test agents (10 and 100 μg/mL) and left for one hour in the incubator before the induction of inflammation. Wells that did not receive any treatment with the samples were considered the control group cells. Directly afterwards, LPS was introduced to all wells whether treated or non-treated group (control) to induce inflammation in cells. The cells were kept in the incubator for 24 h. Finally, the cell culture supernatants were collected, mixed up with Griess reagent in a dim light room at 25 °C, and nitrite absorbance was recorded for each sample at 540 nm.

*COX enzyme inhibition assay.* COX Inhibitor Screening Assay kit (Catalogue # 560131, Cayman ACE™, EIA kits, Ann Arbor, MI, USA) was used to assess the ability of the test agents, as well as the reference drug Indomethacin to interfere with both COX-1 and COX-2 enzymes. Five different logarithmic concentrations (0.01, 0.1, 1, 10, 100 μg/mL) of the test agents were assessed by the kit to establish a dose-response inhibition curve and to compute the half maximal inhibitory concentration (IC_50_, μg/mL) for each sample on COX-1 and COX-2.

*Lipoxygenase (LOX) enzyme inhibition assay.* LOX Inhibitor Screening Assay Kit (catalog #KA1329, Abnova, Taipei, Taiwan) was utilized to detect the ability of the test agents, as well as the reference drug Zileuton to inhibit LOX enzyme. Five different logarithmic concentrations (0.01, 0.1, 1, 10, 100 μg/mL) of the test agents were assessed by the kit to establish a dose-response inhibition curve and to compute IC_50_ for each sample on LOX enzyme.

## 5. Conclusions

We report properties of hydroethanolic extracts prepared from Pinot Noir grape marc (Murfatlar, Romania) in various conditions. Concerning the chemical composition of extracts, the most abundant components were from catechins class, which could explain their good α-glucosidase inhibitory activity close to that of (-)-epigallocatechin gallate. The hydroethanolic PN extracts exerted a very good radical scavenging activity (812.47–856.86 mg TE/g extract–DPPH assay and 651.80–689.09 m mg TE/g extract–ABTS method), except the extract prepared by USAE, although this extract has the highest TP index, but the lowest anthocyanins content.

Loading of PN extract on MCM-NH_2_-Fuc support resulted in enhanced anti-inflammatory activity against COX-2 and LOX enzymes with lower IC_50_ values (0.10 and 4.20 μg/mL, respectively) than for free extract (0.57 and 7.03 μg/mL, respectively) or reference drugs, Indomethacin (0.53 μg/mL) and Zileuton (6.10 μg/mL), respectively. The formulation proposed in this study containing fucoidan coated silica could be used in nutraceutical or cosmetics due to its anti-inflammatory and radical scavenging capacity, while PN@Diatomite material could be useful as fertilizer for valuable plants. We demonstrated that an abundant waste resulting from the winemaking process can be valorized in value-added materials.

## Figures and Tables

**Table 1:** Chemical composition of Pinot Noir extracts evaluated by RP-HPLC.

Compounds	Concentration in Extract (mg_compound_/g_extract_)			
	PN	PN(R)	PN(C)	PN(US)
Gallic Acid	0.791 ± 0.001	0.933 ± 0.001	0.736 ± 0.002	0.820 ± 0.005
Protocatechuic acid	0.337 ± 0.001	0.396 ± 0.002	0.250 ± 0.002	0.278 ± 0.001
Catechin hydrate	14.673 ± 0.018	18.722 ± 0.015	11.505 ± 0.016	12.784 ± 0.004
Vanillic acid	0.687 ± 0.001	0.833 ± 0.002	0.579 ± 0.002	0.640 ± 0.001
Siringic acid	0.928 ± 0.002	1.168 ± 0.002	0.745 ± 0.001	0.913 ± 0.005
(-) Epicatechin	10.613 ± 0.031	13.218 ± 0.008	8.772 ± 0.027	9.700 ± 0.037
Delphinidin	0.401 ± 0.004	0.402 ± 0.003	0.453 ± 0.001	0.080 ± 0.001
Rutin hydrate	0.517 ± 0.001	0.559 ± 0.002	0.523 ± 0.002	0.499 ±0.002

## Data Availability

Data are contained within the article and Supplementary Materials.

## Supplementary Materials

The following supporting information can be downloaded at: https://www.mdpi.com/article/10.3390/molecules29133122/s1. Figure S1. Chromatograms of Pinot Noir extracts; Figure S2. SEM micrograph of PN(R) residue obtained by thermal treatment at 800 °C (A) and its EDX spectrum (B); Figure S3. N_2_ adsorption-desorption isotherms for pristine MCM-41 and MCM-41 functionalized with amine groups (A) and their corresponding pore size distribution curves determined with DFT (B).
