# Supplementary material for: Anti-Inflammatory, Antidiabetic, and Antioxidant Properties of Extracts Prepared from Pinot Noir Grape Marc, Free and Incorporated in Porous Silica-Based Supports
Source: Molecules. 2024 Jun 30;29(13):3122. doi: 10.3390/molecules29133122 (PMC11243692; doi:10.3390/molecules29133122)
Supplement: Supplementary file 1 [file molecules-29-03122-s001.zip › molecules-3058521-supplementary.pdf]

# Anti-Inflammatory, Antidiabetic, and Antioxidant Properties of Extracts Prepared from Pinot Noir Grape Marc, Free and Incorporated in Porous Silica-Based Supports

**Mihaela Deaconu [1,2], Anil Abduraman [1], Ana-Maria Brezoiu [1], Nada K. Sedky [3], Simona Ioniță [1], Cristian Matei [1,*], Laila Ziko [3] and Daniela Berger [1,*]**

[1] Faculty of Chemical Engineering and Biotechnologies, National University of Science and Technology Politehnica Bucharest, 1-7 Gheorghe Polizu Street, 011061 Bucharest, Romania; mihaela.deaconu@upb.ro (M.D.); ana_maria.brezoiu@upb.ro (A.-M.B.); simona.ionita05@gmail.com (S.I.)
[2] CAMPUS Research Institute, National University of Science and Technology Politehnica Bucharest, 060042 Bucharest, Romania
[3] Department of Biochemistry, School of Life and Medical Sciences, University of Hertfordshire, Hosted by Global Academic Foundation, R5 New Garden City, New Administrative Capital, Cairo 11835, Egypt; nadasedky22@gmail.com (N.K.S.); l.adel@gaf.edu.eg (L.Z.)
* Correspondence: cristian.matei@upb.ro (C.M.); daniela.berger@upb.ro (D.B.)

The phenolic compounds of the extracts were determined by reverse phase high-performance liquid chromatography (HPLC; Shimadzu Nexera 2, Shimadzu Corporation, Kyoto, Japan) with a photodiode array detector (SPD-M30A, Shimadzu Corporation, Kyoto, Japan) in the range of 250–600 nm based on their retention time and the spectrum similarity with the standard compounds. The compounds quantification was performed based on the calibration curve of each standard compound.

In HPLC analyses were used the following standard substances: gallic acid (98%, Alfa Aesar, Ward Hill, MA, USA), protocatechuic acid (>98%, HPLC, TCI, Tokyo, Japan), caftaric acid (Molekula GmbH, Munich, Germany), vanillic acid (>98%, GC, TCI, Tokyo, Japan), syringic acid (>98.5%, Molekula GmbH, Munich, Germany), caffeic acid (98%, HPLC, Sigma, Merck Group, Darmstadt, Germany), *trans*-p-coumaric acid (analytical standard, Sigma-Aldrich Co. Merck Group, Darmstadt, Germany), trans-ferulic acid (>98%, GC) and chicoric acid (>98%) from TCI, Tokyo, Japan), chlorogenic acid (primary reference standard, HWI group, Alpen Aan de Rijn, The Netherlands), rosmarinic acid (>98%, HPLC, Sigma, Merck Group, Darmstadt, Germany), catechin hydrate (>98%, HPLC, Sigma, Merck Group, Darmstadt, Germany), (−) epicatechin (>98%, HPLC, TCI, Tokyo, Japan), quercetin (>95%, HPLC), rutin hydrate (95%, HPLC), myricetin (>96%, HPLC-grade), and kaempferol (>97%, HPLC) from Sigma (Merck Group, Darmstadt, Germany), ellagic acid dihydrate (>98%, HPLC, TCI, Tokyo, Japan), trans-resveratrol (certified reference material, Sigma-Aldrich Co. Merck Group, Darmstadt, Germany), anthocyanidins: cyanidin chloride (>95%, HPLC, Sigma, Merck Group, Darmstadt, Germany), malvidin chloride (>95%, HPLC, Sigma-Aldrich Co. Merck Group, Darmstadt, Germany), pelargonidin chloride (Aldrich Chemical Co Inc., Milwaukee, WI, USA) and delphinidin chloride (analytical standard, Sigma-Aldrich Co. Merck Group, Darmstadt, Germany). For mobile phases ethanol and acetonitrile were purchased from Riedel-de Haën, Honeywell Riedel-de Haën, Seelzer, Germany, and formic acid from Merck (Merck Group, Darmstadt, Germany). For all solutions and experiments was used ultrapure water produced with Millipore Direct-Q3 ultraviolet (UV) water purification system with Biopack UF cartridge (Millipore, Merck Group, Darmstadt, Germany).

For obtaining mesoporous silica-type supports, tetraethyl orthosilicate (TEOS, Fluka, Seelzer, Germany), cetyltrimethylammonium chloride (25 wt. % in $H_2O$, CTAC, Sigma-Aldrich Co. Merck Group, Darmstadt, Germany), triethanolamine (98%, Sigma-Aldrich), $NH_4Cl$ (Sigma-Aldrich), poly(ethylene glycol) methyl ether (PEG, average Mn 550, Sigma-Aldrich), (3-aminopropyl)triethoxysilane (APTES, 99%, Sigma-Aldrich), toluene (Carlo Erba), and fucoidan (Sigma-Aldrich) were used as received.

For UV-vis analyses were used the following reagents: sodium carbonate, potassium persulphate, Folin–Ciocalteu reagent, 2,2-diphenyl-1-picrylhydrazyl (DPPH), and 2,2′-azino-bis (3 ethylbenzothiazoline-6-sulphonic acid) (ABTS) purchased from Sigma-Aldrich (Sigma-Aldrich Co. Merck Group, Darmstadt, Germany) and 6-hydroxy- 2,5,7,8-tetramethylchroman-2-carboxylic acid from Aldrich (Trolox, 97%, Aldrich Chemical Co Inc., Milwaukee, WI, USA). For antidiabetic activity via $\alpha$-glucosidase inhibitory assay, $\alpha$-glucosidase from Saccharomyces cerevisiae (Sigma-Aldrich) solution (0.5 U/mL in phosphate buffer solution, PBS, pH 6.8) incubated at 37 °C for 15 min and *p*-nitrophenol-$\alpha$-D-glucopyranoside from Sigma-Aldrich (5 mM in PBS pH 6.8), as well as (–)-epigallocatechin gallate, EGCG (from Extrasynthese, Lyon, France) as reference compound were used.

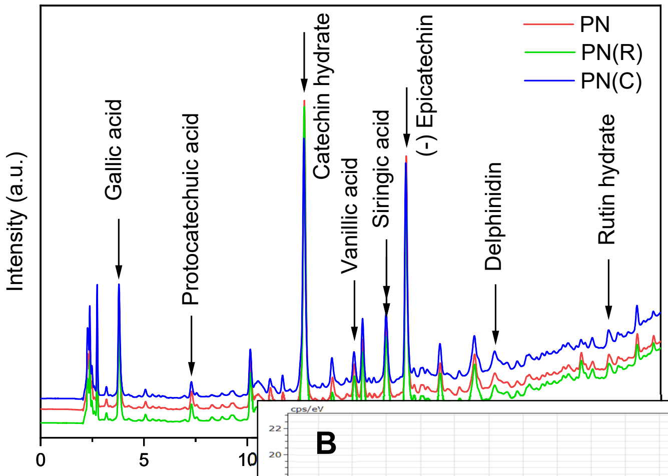


Figure S1. Ch

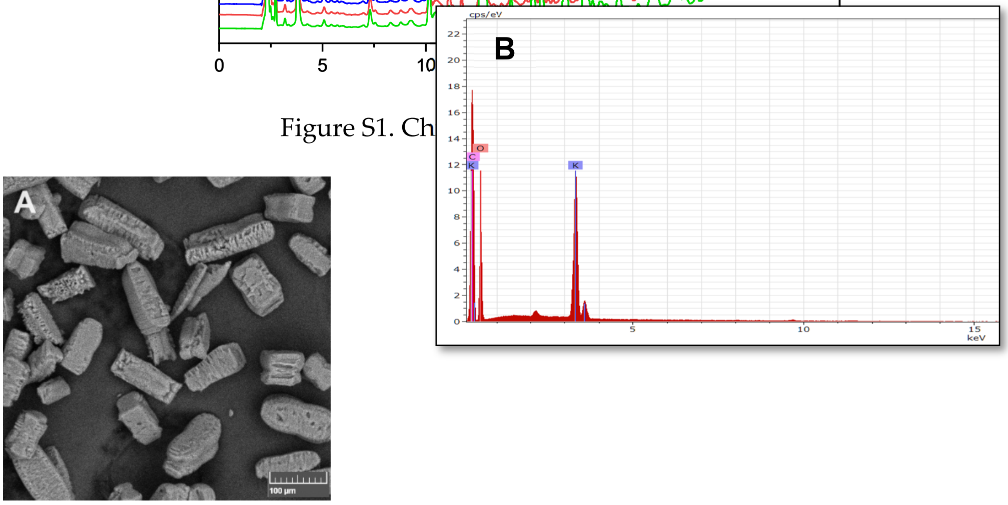


Figure S2. SEM micrograph of PN(R) residue obtained by thermal treatment at 800ºC (A) and its EDX spectrum (B).

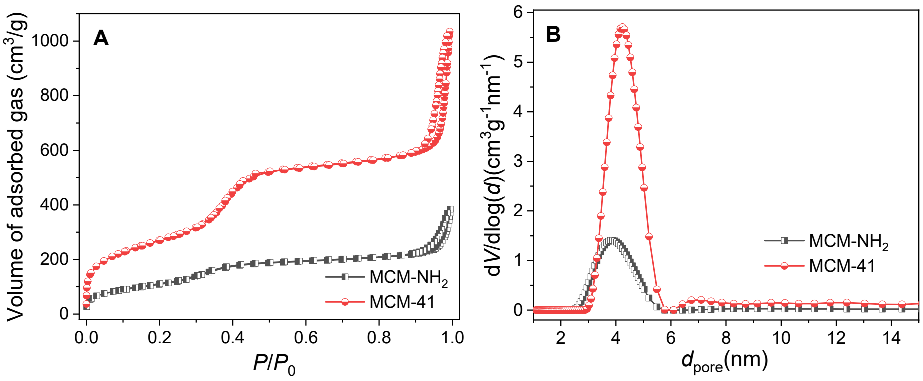


Figure S3. $N_2$ adsorption-desorption isotherms for pristine MCM-41 and MCM-41 functionalized with amine groups (A) and their corresponding pore size distribution curves determined with DFT.
